# Supplementary material for: Implementation of the EIRA 3 Intervention by Targeting Primary Health Care Practitioners: Effectiveness in Increasing Physical Activity
Source: Int J Environ Res Public Health. 2021 Oct 8;18(19):10537. doi: 10.3390/ijerph181910537 (PMC8507856; doi:10.3390/ijerph181910537)
Supplement: Supplementary file 1 [file ijerph-18-10537-s001.zip › ijerph-1386479-supplementary.pdf]

Table S1. Description of the intervention for each of the unhealthy behaviours and the stage of change of participants.

	Unhealthy behaviours								
	Tobacco use			Physical inactivity			Non-adherence mediterranean diet		
**Stage of change**	**Individual**	**Group**	**Community**	**Individual**	**Group**	**Community**	**Individual**	**Group**	**Community**
Precontemplation	**Very brief intervention + SMS**			Very brief intervention + SMS			Very brief intervention + SMS		
Contemplation			Social prescribing	Brief intervention App+ SMS	Health education workshops	Social prescribing	Brief intervention App+ SMS	Health education workshops	Social prescribing
Preparation	Brief intervention + SMS								
Action				Very brief intervention + SMS					
Maintenance	Very brief intervention + SMS						Very brief intervention + SMS		
Termination									

Table S2. Community physical activity resources in participants PHCs by regions.

**Region**	**Resources (N)**	**Physical activity**	**Intensity**
Andalusia	6	gymnastics, pilates, dance, zumba, aerobics	moderate, high
Aragon	8	walking, cycling, swimming	low, moderate, high
Balearic Island	20	gymnastics, pilates, yoga, fitness, swimming, aquagym, Tai-chi, dancing, 'solidarity garden'	low, moderate
Basque Country	11	walking, gymnastics, swimming, aquagym, dance, 'healthy aging'.	moderate, high
Castile and Leon	1	walking	low, moderate
Catalonia	11	aerobics, fitness, walking, dancing	low, moderate, high
Galicia	21	gymnastics, pilates, yoga, swimming, aquagym, Tai-chi, dancing, zumba, cycling	low, moderate, high
